# Metabolic profiling reveals therapeutic effects of *Herba Cistanches *in an animal model of hydrocortisone-induced 'kidney-deficiency syndrome'

**DOI:** 10.1186/1749-8546-3-3

**Published:** 2008-03-10

**Authors:** Yunping Qiu, Minjun Chen, Mingming Su, Guoxiang Xie, Xin Li, Mingmei Zhou, Aihua Zhao, Jian Jiang, Wei Jia

**Affiliations:** 1 School of Pharmacy, Shanghai Jiao Tong University, Shanghai 200030, China; 2 Shanghai University of Traditional Chinese Medicine, Shanghai 201203, China; 3 Shanghai Center for Systems Biomedicine, Shanghai Jiao Tong University, Shanghai 201203, China

## Abstract

**Background:**

*Herba Cistanches *(*Roucongrong*) is effective in treating *Shenxu Zheng *('kidney-deficiency syndrome'). However, the mechanisms and systemic metabolic responses to the herbal intervention are unclear.

**Methods:**

Using GC-MS-based metabolic profiling, we investigated the metabolic responses to *Herba Cistanches *intervention in a rat model of the hydrocortisone-induced 'kidney-deficiency syndrome'.

**Results:**

The metabolic profiles of the rats after hydrocortisone injection deviated from the pre-dose metabolic state at different time points, ranging from day 1 to day 10, whereas the metabolic profiles of the rats treated with both hydrocortisone and water extract of *Herba Cistanches *returned to the pre-dose state on day 10.

**Conclusion:**

The intervention of *Herba Cistanches *caused a systemic recovery from the hydrocortisone-induced metabolic perturbation in rats. This study also demonstrates that metabolic profiling is useful in studying therapeutic mechanisms of herbal medicines.

## Background

Through modulation of biochemical reactions, control mechanisms, and enzyme activities, many drugs or chemicals cause fluctuation of metabolites present in single cells, tissues or body fluids [1]. Metabolic profiling, *i.e*. probing metabolites of low molecular weight (MW < 1000 Da) by means of advanced analytical instrument coupled with multivariate statistics, can show systemic responses of living systems to xenobiotics. It is also technically feasible to catalogue all the multifactorial heritable and environmentally influenced metabolic profiles of an organism, including the physiopathological consequences of toxin and/or disease-induced disturbances or disequilibria in metabolic regulatory network on a systemic level. To date, metabolic profiling has been established in screening, diagnosis, prognosis of diseases [2-4], and safety evaluation of certain drugs and chemicals [5-11].

Nuclear magnetic resonance (NMR) [12] and mass spectrometry (MS) [13], applied alone or in combination, have been used in profiling and characterizing metabolic consequences of toxin and/or disease-induced disturbances. NMR, which does not require tedious sample preprocessing, is a fast and simple method to obtain intrinsic information from complex and intact biological samples. On the other hand, the wide application of hyphenated MS in metabolic profiling is due to its high sensitivity and availability [14,15]. Notably, GC-MS-based metabolic profiling has been used in discovering mechanisms of drugs and herbicides *in vivo*, biomarkers of diseases [16] and effects of altered gene expression on metabolism, and monitoring performance of organisms in biotechnological applications [17-20].

*Herba Cistanches *(*Roucongrong*), a common Chinese tonic herb which grows in desert, exhibits marked activities for improving memory [21] and/or sexual potency [22], free radical scavenging, anti-aging [23-26] and neuroprotection [27,28]. For centuries, *Herba Cistanches *has been effectively used in treating *Shenxu Zheng *('kidney-deficiency syndrome') [29]. Recently *Herba Cistanches *was demonstrated to have ameliorated hydrocortisone-induced kidney disorders [30]; however, its metabolic consequences are not clear. Our previous study [31] found that metabolic profiles of rats exposed to hydrocortisone at a high dosage *(i.e*. an animal model for the 'kidney-deficiency syndrome') [32], showed a unique biochemical pattern of endogenous metabolites in urine. These results inspired us to study the mechanisms of consistent biochemical changes following hydrocortisone modification, using GC-MS-based metabolic profiling to investigate whether *Herba Cistanches *could reverse or counteract aberrant metabolic effects of hydrocortisone.

## Methods

### Materials and instrument

*Herba Cistanches *was purchased from Shanghai Leiyunshang Pharmaceutical Co Ltd (China) and identified as *Cistanche deserticola *Y C Ma by Dr Mengyue Wang (Laboratory of Pharmacognostics, School of Pharmacy, Shanghai Jiao Tong University) according to a standard protocol [33]. Hydrocortisone solution for injection (0.5%) was purchased from Shanghai Xinyi Pharmaceutical Co (China). The derivatizing reagents were N-methyl-N-trimethylsilyltrifluoroacetamide (MSTFA) (Sigma-Aldrich Inc, USA) and Trimethyliodosilane (TMSI) (Sigma-Aldrich Inc, USA) mixed at a ratio of 1000:1. All reagents used in the experiment were of analytical grade. Ultra-pure water was prepared with Millipore purification system (18.2 MΩ, USA). Metabolic cages were purchased from Suzhou Fengshi Laboratory Animal Experiment Co Ltd (China).

### Preparation of Herba Cistanches extract

Five hundred grams of coarsely pulverized plant material was refluxed with 2 L of ultra-pure water for 2 hours. After filtration, the extract was evaporated to about one tenth of the original volume on a Buchi rotary evaporator and was diluted to 250 mL in a volumetric flask with ultra-pure water. The final concentration of crude *Herba Cistanches *extract was 2 g/mL.

### Dosage and sampling

The handling of all animals in this study conformed to the national guidelines and was performed at the Center for Laboratory Animals, Shanghai University of Traditional Chinese Medicine, Shanghai, China. A total of 19 nine-week-old male Wistar rats were purchased from Shanghai Laboratory Animal Co Ltd (China). All animals were kept at a barrier system with regulated temperature (20–22°C) and humidity (60 ± 10%), and on a 12 hour dark/light cycle with lights on at 8:00 am. The rats were given *ad libitum *food and water. After two weeks' acclimation, the animals were transferred to individual metabolic cages and randomly divided into three groups: (1) treatment group (n = 7) in which hydrocortisone (5%) was injected i.p. at 1.5 mg/100 g of body weight followed by oral administration of *Herba Cistanches *extract for 10 days; (2) model group (n = 7) in which hydrocortisone (5%) was injected i.p. at 1.5 mg/100 g once daily for 10 days; and (3) control group (n = 5) in which the vehicle was injected i.p. at about 0.6 mL for 10 days [31]. *Herba Cistanches *was administered to the treatment group at a dose of 20 g/kg following the recommendation by Shen *et al*. [30]. Twenty-four hour urine samples were collected at specific time intervals: pre-dose (-24 – 0 h), day 1 (0 – 24 h), day 3, day 7, and day 10. All the urine samples were centrifuged (6383 × *g*, LG 16-W, Beijing Jingli Centrifuge Co Ltd, China) for 10 minutes to remove suspended debris and immediately stored at -80°C for subsequent GC-MS analysis.

### Sample preparation and GC-MS

GC-MS was performed according to our previous study with minor modifications [31]. Briefly, each 0.5 μL aliquot of trimethylsilyl (TMS) derivatized analyte was injected into a fused-silica capillary column (17 m × 220 μm inside diameter, 0.11 μm film thickness; HP Ultra-1, Agilent J&W Scientific, USA). GC-MS was conducted on a hyphenated PerkinElmer gas chromatograph and TurboMass-Autosystem XL mass spectrometer (PerkinElmer Inc, USA).

### Data processing and multivariate analysis

The GC-MS data were converted into NetCDF format through DataBridge (PerkinElmer Inc, USA). Custom scripts were run in MATLAB 7.0 (The MathWorks Inc, USA) to perform baseline correction, peak deconvolution and alignment, internal standard exclusion, and normalization to the total sum of the chromatogram. The resultant 3-dimensional matrix encompassing arbitrary peak index (paired retention time-*m/z*), samples (observations), and normalized peak areas (variables) were imported into the SIMCA-P 11.0 software package (Umetrics, Sweden) for multivariate analysis.

Mean-centering was performed column-wise to remove the offsets. All the measured metabolites were treated on an equal level with auto-scaling (scaled to unit variance) prior to multivariate analysis. Principal component analysis (PCA) was performed using the SIMCA-P 11.0 software to reveal the general clustering, grouping, and trends among the subjects without prior knowledge. The first principal component (PC1) represents the most variance in the data. The second principal component (PC2) is orthogonal to PC1, and represents maximum amount of variance not explained by PC1. The remaining principal components were constructed in the same manner. Meanwhile, mean trajectories of PCA scores were used to provide a dynamic indication for the onset, progression, and/or recovery of the syndrome through time. Correlation coefficients from the partial least squares – discriminant analysis (PLS-DA) were used to rank the importance of each variable to further capture the differentially expressed metabolites accountable for the separation between groups. PLS-DA is derived from the partial least squares (PLS) method which is a generalized multiple regression method dealing with multiple collinear predictor and response variables [34]. The PLS-DA was conducted using the SIMCA-P 11.0 software [35]. A typical 7-round cross-validation was performed. One seventh of the samples were excluded from the model in each round so as to validate the model. This procedure was repeated in an iterative manner for cross-validation until each sample had been excluded once.

### Univariate analysis

The differentially expressed metabolites identified from multivariate analysis were also verified in the MATLAB 7.0 software (The MathWorks Inc, USA) by nonparametric Kruskal-Wallis test with a significance level at P < 0.05.

## Results and Discussion

### Interpretation of GC-MS spectra

**Figure 1:**
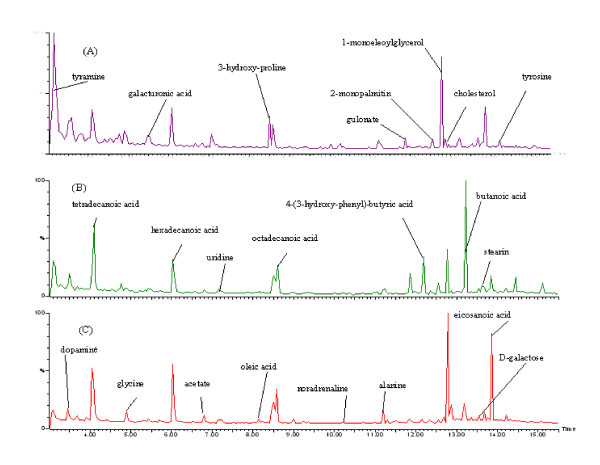
Typical GC-MS total ion current (TIC) chromatograms of urine on day 10 from the treatment group (A), model group (B) and control group (C).

Typical GC-MS total ion current (TIC) chromatograms of rat urine on day 10 from the treatment group, model group and control group are shown in Figure 1. Using our optimized GC-MS analysis protocol in association with a software-based peak deconvolution procedure, a total of 117 individual metabolites were consistently detected in at least 90% of the urine samples. Compound identification of peaks of interest was performed by comparing the mass spectral fragment against the NIST (National Institute of Standards and Technology) reference libraries, Wiley libraries and reference standards. We were able to verify 23 out of the 117 metabolites (20%), the majority of which were amino acids, polyamines, fatty acids, purines and adrenal hormones that are mainly involved in energy metabolism, lipid metabolism and amino acid metabolism.

### Time-dependent changes in urine samples

**Figure 2:**
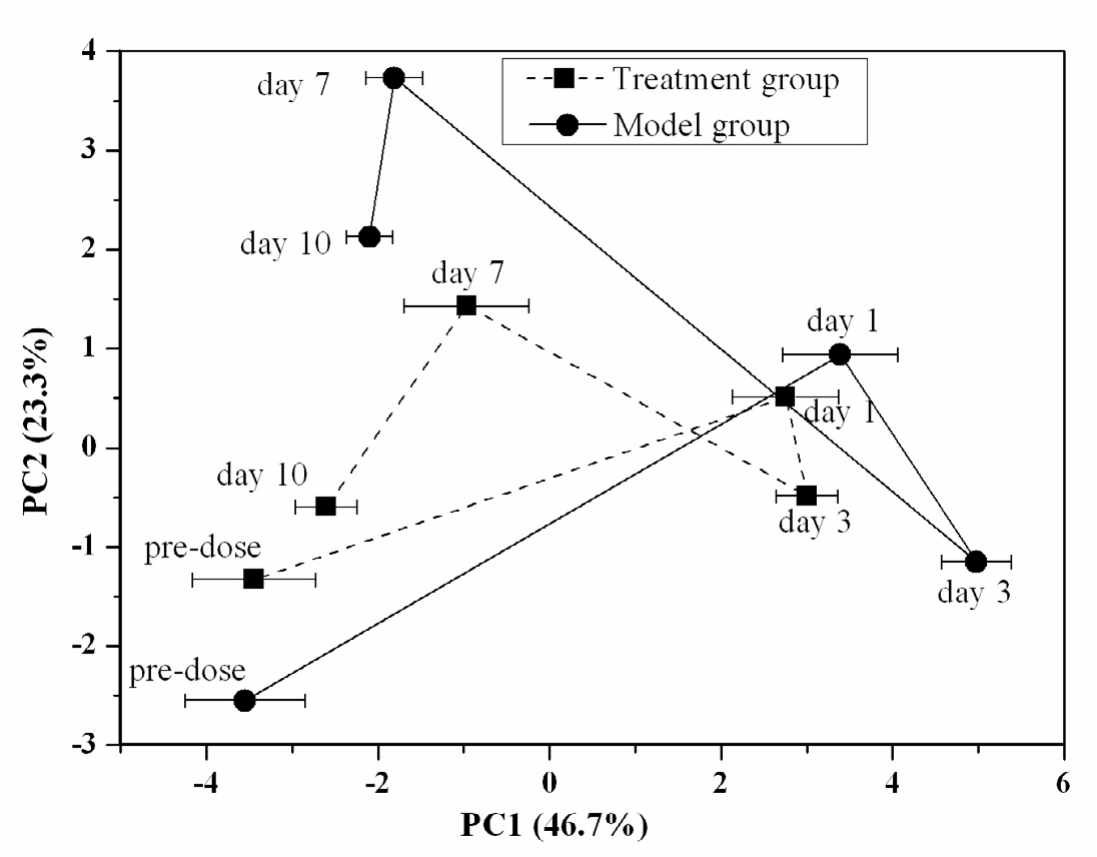
**Mean trajectory of PC1 *vs *PC2 scores for urine samples from the model group (-●-) and treatment group (--●--). **Each dot denotes a mean score at different time points, *i.e*. pre-dose, days 1, 3, 7 and 10. Error bar represents the standard deviation for each time point obtained by the first principal component.

The mean trajectories of the PCA scores derived from the model group and treatment group were illustrated in Figure 2. Transient shift in the trajectory plot revealed the dynamic progress of the 'kidney-deficiency syndrome' induced by hydrocortisone alone or in combination with *Herba Cistanches *treatment. In the model group, metabolic patterns on day 1 and day 3 were different from those on day 7 and day 10, suggesting that the metabolic regulatory network on day 1 and day 3 might have undergone a transient period with high fluctuations, and that the perturbed network might have been restored on day 7 and day 10 which ultimately led to a stable pattern close to the pre-dose state. Analogously, the fact that metabolic pattern of day 1 and day 3 obviously deviated from that of pre-dose in the treatment group indicated the dominant 'kidney-deficiency syndrome' state. In this period, the effects of hydrocortisone were probably dominant over those of the *Herba Cistanches *extract. These findings were consistent with the general observation that rats from both groups showed less activity on day 1 and day 3. Interestingly, the metabolic patterns on day 7 and day 10 gradually and significantly approached the pre-dose state, suggesting that *Herba Cistanches *had some counteracting or therapeutic effects on the rats exposed to hydrocortisone. These results support the clinical findings that *Herba Cistanches *is effective in treating the 'kidney-deficiency syndrome'. In general, both trajectories provide a visual, overall and dynamic picture of the onset, progression and recovery of the 'kidney-deficiency syndrome'.

### Comparative metabolic analysis of urine samples

**Figure 3:**
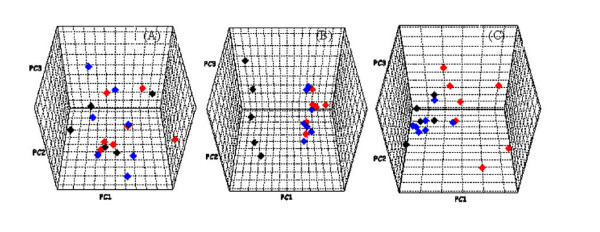
**Comparison of metabolic profiles from the control group (black diamond), model group (red diamond), and treatment group (blue diamond) at different time points: pre-dose (A), day 3 (B) and day 10 (C).** Each dot in the PCA scores plot represents the data obtained from a rat.

To better understand the metabolic effects of hydrocortisone, we compared the metabolic profiles obtained from the control, model and treatment groups. General clustering of the three groups can be readily observed at various time points, *i.e*. pre-dose, day 3 and day 10 (Figure 3). While there is no separation tendency in pre-dose urine profiles, the metabolic profiles deviated from those of the control group on day 3 after hydrocortisone exposure. The metabolic perturbation by hydrocortisone appeared in both the model and treatment groups. However, after a consecutive 7-day treatment with *Herba Cistanches*, metabolic profiles of the treatment group became comparable to those of the control group again, indicating that *Herba Cistanches *did effectively restore the perturbed metabolism.

### Differential identification of metabolic profiles

**Figure 4:**
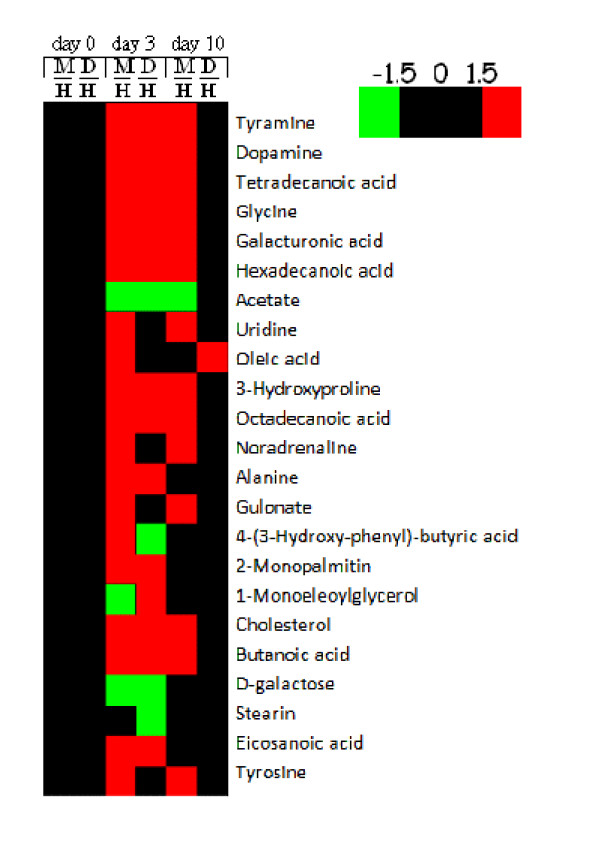
**Fold changes of the key metabolites.** Red color denotes relative elevated concentrations (fold changes > 1.5), whereas green color denotes relative reduced concentrations (fold changes < -1.5). Fold changes ranging from -1.5 to 1.5 are considered to be physiological variations. A fold change (M/H, D/H) is the concentration ratio of the model group or treatment group to the control group.

A cross-validated PLS-DA model was used to identify the key metabolites in different metabolic profiles for easier differentiation between the control group rats and model group (*i.e*. hydrocortisone-induced) rats with or without *Herba Cistanches *treatment on day 3 (Table 1). Fold changes in relative concentration of each key metabolite between groups were determined and the corresponding visualization of the changes between groups at pre-dose, day 3 and day 10 was produced (Figure 4). As shown in Figure 4 and Table 1, while most of the endogenous metabolites significantly increased or decreased in the model group, those in the treatment group underwent a transient period as observed on day 1 and day 3 and gradually approached the control (normal) level. For example, as compared to the slight variation (1.1–1.5 times) of the metabolites in the treatment group on day 10, the greatly enhanced levels (1.7–3.0 times) of urinary tyrosine, tyramine, dopamine, and noradrenaline were observed in the model group throughout the experiment. Our previous study showed that the enhanced catecholamine metabolism induced by glucocorticoids resulted in over-consumption of immune functions, thereby leading to the 'kidney-deficiency syndrome' [31]. *Herba Cistanches*, a tonic herb which improves the immune system [36], may counteract against some effects of hydrocortisone. *Herba Cistanches *may also be able to restore a normal metabolic regulatory network. Further experiments with different approaches such as molecular biology, cell biology and plant chemistry are required to delineate the actions of *Herba Cistanches *(and its constituents) in the 'kidney-deficiency syndrome'.

**Table 1:** A list of metabolites included in the metabolic profiling of the present study

**Compounds**	**M/H/0**	**D/H/0**	**M/H/3**	**D/H/3**	**kw (P)**	**corr coeffs**	**M/H/10**	**D/H/10**
Tyramine	1.1	1.1	2.8	2.8	0.0074	0.66	2.1	1.2
Dopamine	-1.5	1.0	3.0	2.8	0.0045	0.88	1.7	1.4
Tetradecanoic acid	1.2	1.2	3.0	3.0	0.0045	0.65	3.0	1.0
Glycine	1.4	-1.3	2.8	2.1	0.0074	0.77	1.6	1.3
Galacturonic acid	1.4	1.3	3.0	3.0	0.0045	0.88	1.8	1.5
Hexadecanoic acid	-1.0	1.2	2.2	2.5	0.0118	0.58	2.1	1.0
Acetate	-1.4	-1.3	-3.0	-3.0	0.0045	-0.71	-2.6	-1.1
Uridine	1.4	1.4	2.5	1.3	0.0045	0.67	1.8	1.1
Oleic acid	-1.1	-1.1	2.2	-1.1	0.0118	0.67	1.2	1.8
3-Hydroxyproline	-1.1	1.0	2.0	1.6	0.0284	0.64	2.9	1.3
Octadecanoic acid	1.3	-1.3	2.5	2.5	0.0045	0.79	1.8	1.1
Noradrenaline	-1.4	-1.3	2.5	1.0	0.0045	0.77	2.8	1.1
Alanine	1.0	1.0	2.5	2.2	0.0045	0.67	1.5	1.0
Gulonate	-1.1	1.2	1.9	1.3	0.0424	0.53	2.8	1.4
4-(3-Hydroxy-phenyl)-butyric acid	1.2	1.3	2.1	-1.7	0.0424	0.86	1.1	1.1
2-Monopalmitin	-1.3	-1.3	2.5	2.4	0.0045	0.73	1.2	1.2
1-Monoeleoylglycerol	1.1	1.1	-1.9	1.6	0.2912	0.6	-1.3	-1.3
Cholesterol	1.3	1.3	2.5	2.5	0.0045	0.62	1.7	1.0
Butanoic acid	1.1	-1.2	3.0	3.0	0.0045	0.83	1.9	1.1
D-galactose	-1.4	-1.4	-1.8	-1.8	0.0618	-0.6	-1.3	-1.3
Stearin	-1.0	1.2	-1.3	-1.6	0.4649	0.52	-1.4	-1.5
Eicosanoic acid	-1.0	1.1	1.8	1.9	0.0618	0.85	1.4	1.1
Tyrosine	1.3	-1.0	2.8	1.2	0.0074	0.88	2.3	1.4

Note: The correlation coefficients (corr coeffs) of all compounds were calculated from a cross-validated PLS-DA model (Q^2^Y_cum _= 0.899, a satisfactory model using two components) on day 3 between the control group and model group with or without *Herba Cistanches *treatment. Additionally, the fold changes were tested by the nonparametric Kruskal-Wallis test. Kw (P) denotes the P values of the test. H = control group, M = model group, D = treatment group. For example, H/M/0 represents the relative fold changes (model to control) at the pre-dose state.

## Conclusion

The present metabolic profiling study using GC-MS showed that *Herba Cistanches *caused a systemic recovery from the hydrocortisone-induced metabolic perturbation in rats, an animal model for the 'kidney-deficiency syndrome'. This study also demonstrates that metabolic profiling is a useful method to study therapeutic effects of herbal medicines.

## Abbreviations

MS: mass spectrometry; GC-MS: gas chromatography-mass spectrometry; NMR: nuclear magnetic resonance; PCA: principal component analysis; PLS-DA: partial least squares – discriminant analysis

## Competing interests

The author(s) declare that they have no competing interests.

## Authors' contributions

YQ and MC performed the animal and instrumental experiments and drafted the manuscript. MS and GX analyzed the data and helped draft the manuscript. XL helped with animal experiments. MZ and AZ prepared *Herba Cistanches *extract. JJ provided suggestions for the experiment design and the manuscript. WJ supervised all the experiments and revised the manuscript. All authors read and approved the final manuscript.
